# Platelet-rich fibrin as a therapeutic modality for oroantral communication closure: a systematic review and meta-analysis

**DOI:** 10.3389/froh.2026.1759248

**Published:** 2026-03-11

**Authors:** Manar Kh Almutiri, Nawaf Ahmad Almutairi, Abdurrahman Saud Almutairy, Ahmed Adnan Alwosaibai, Ahmed Alruwaili, Faris Sultan Al-Mutairi, Reema Hasan Almutairi, Sarah Khalid Alsaairi, Hassan Albader, Isra Aljazeeri

**Affiliations:** 1 Ministry of Health, Kuwait City, Kuwait; 2 Farwaniya Hospital, Kuwait City, Kuwait; 3 Ministry of Health, Madinah, Saudi Arabia; 4 College of Medicine, Qassim University, Buraydah, Saudi Arabia; 5 Department of General Surgery, Alqurayat General Hospital, Alqurayat City, Saudi Arabia; 6 College of Medicine, Taibah University, Madina, Saudi Arabia; 7 College of Medicine, Tabuk University, Tabuk, Saudi Arabia; 8 Collage of Medicine, King Khalid University, Abha, Saudi Arabia; 9 King Faisal General Hospital, Ministry of Health, Hofuf, Saudi Arabia; 10 Department of Otolaryngology-Head and Neck Surgery, College of Medicine, King Saud University, Riyadh, Saudi Arabia; 11 King Abdullah Ear Specialist Center (KAESC), King Saud University Medical City, Riyadh, Saudi Arabia; 12 Aljaber Ophthalmology and Otolaryngology Specialized Hospital, Ministry of Health, Ahsa, Saudi Arabia

**Keywords:** buccosinusal communication, Fibrin mesh, oroantral communication, oroantral fistula, platelet-rich fibrin (PRF)

## Abstract

**Background:**

Oroantral communication (OAC) is an abnormal connection between the oral cavity and maxillary sinus, often occurring after posterior maxillary tooth extraction. If untreated, OAC can progress to oroantral fistulas (OAFs), leading to chronic sinusitis and treatment failure. Platelet-rich fibrin (PRF), an autologous biomaterial with regenerative potential, has been proposed as a therapeutic option. This study aimed to evaluate the efficacy and safety of PRF in the closure of OACs/OAFs.

**Methods:**

This systematic review and meta-analysis followed PRISMA guidelines and was registered with PROSPERO. A comprehensive literature search was conducted in April 2025 across Cochrane, PubMed, Google Scholar and Web of Science. Studies were selected using PICO criteria: patients with OACs/OAFs; PRF as a standalone or adjunct intervention; comparison to conventional treatments; outcome of successful closure; and human studies published in English or Arabic. Data extraction and risk of bias assessment were performed independently by multiple reviewers using Cochrane RoB 2, MINORS, and MMS tools. Meta-analysis was conducted using Review Manager software.

**Results:**

Nineteen studies including 442 patients were included. PRF demonstrated high closure rates, often within three weeks, with enhanced mucosal and bone healing. Minimal complications were reported. Although methodological heterogeneity and moderate-to-high risk of bias were observed, the overall findings support the beneficial role of PRF in OAC/OAF management, particularly in minor to moderate defects.

**Conclusion:**

PRF is a safe, effective, and minimally invasive adjunct for the closure of OACs/OAFs. Its application is associated with significantly reduced patient morbidity, including less pain, lower analgesic consumption, minimal swelling, and greater overall comfort. Furthermore, as a minimally invasive and entirely autologous material, PRF eliminates the risks of immunogenic reactions or disease transmission, while its application helps preserve critical anatomical structures like the buccal sulcus, which is essential for future prosthetic rehabilitation.

**Systematic Review Registration:**

PROSPERO CRD420251028634.

## Introduction

Oroantral communication (OAC) refers to an abnormal connection between the oral cavity and the maxillary sinus. It most commonly occurs after the extraction of maxillary posterior teeth, although other causes include maxillary cysts, tumors, and trauma.

OACs require closure to prevent fluids, food particles, and oral bacteria from entering the maxillary sinus. Therefore, the preferred approach after an OAC forms is prompt surgical closure. Early intervention reduces the risk of oroantral fistula (OAF), chronic sinusitis, and treatment failure.

Buccal advancement flaps, palatal rotational flaps, and buccal pad fat grafts are commonly used surgical techniques.

Recent studies suggest that platelet-rich fibrin (PRF) may serve as an effective treatment option for closing defects measuring up to 5 mm in diameter. PRF is a second-generation platelet concentrate composed of a three-dimensional fibrin matrix with a polymerized molecular structure; it contains various blood components, including leukocytes, erythrocytes, platelets, growth factors, and circulating stem cells. The PRF membrane promotes tissue regeneration by stimulating the activity of osteoblasts, gingival fibroblasts, pulp cells, and periodontal ligament cells (1–5).

Given the potential morbidity of conventional techniques, there is growing interest in minimally invasive, biologically active alternatives like PRF, which may promote primary healing and reduce surgical complexity.The aim of this study was to assess the effects of platelet rich fibrin therapy compared to alternative therapeutic modalities in individuals diagnosed with OACs or OAFS, irrespective of age, gender, or etiology, in achieving a successful closure of OACs/OAFs.

Specifically, this study evaluates the overall effectiveness and success rates of platelet-rich fibrin (PRF) in closing oroantral communications (OACs) and fistulas (OAFs). Furthermore, it seeks to compare PRF therapy with other surgical and non-surgical methods, identify associated complications, and assess its impact on healing outcomes and recovery time. Finally, the review will evaluate the consistency of PRF success across diverse clinical settings and patient populations.

## Methods & materials

### Literature review

This review followed the PRISMA (Preferred Reporting Items of Systematic Reviews and Meta- Analysis) model to ensure that studies were selected with the least amount of bias (6). The study protocol was registered with PROSPERO (ID: CRD420251028634). Ethical approval was not required due to the nature of the study. In April 2025, we conducted a systematic search using the following databases: Cochrane, PubMed, Google Scholar, and Web of Science. Additional articles were identified from the bibliographies of reviewed articles. The search utilized the keywords: (Oroantral communication OR Oroantral fistula OR Buccosinusal communication) AND (Platelet-rich fibrin OR PRF OR Fibrin mesh). (Supplementary Table S1) Studies were considered for the review based on PICOS (population, intervention, comparison, outcome, timing, setting) criteria.

### Methodology for selecting studies

For inclusion, the studies had to meet the following criteria: (1) published without time frame limitations; (2) reported individuals diagnosed with OACs or OAFS, irrespective of age, gender, or etiology; (3) reported application of PRF as a primary treatment or in bi- or tri-laminar techniques for OAC/OAF closure; (4) reported outcomes pertinent to the clinical questions; and (5): were RCTs, cohort studies, observational studies, comparative studies, case reports, case series, and multi-center studies published in English or Arabic.

Exclusion criteria were: (1) patients without confirmed OACs/OAFs or those with conditions unrelated to maxillary sinus-oral cavity communications; (2) studies not employing PRF as primary or adjunctive treatment for OACs/OAFs; (3) studies lacking a comparative group or alternative treatment modality where applicable; (4) studies not reporting outcomes related to closure success, postoperative morbidity, or tissue healing; and (5) experimental laboratory studies, animal studies, literature reviews, duplicate publications, books/ book chapters, letters to editor, and commentaries.

### Process of screening and data extraction

Four independent reviewers screened the papers by the title and abstract using the Rayyan search application for systematic reviews (7). Subsequently, the full text of the articles was then reviewed by two independent reviewers simultaneously, with any discrepancy being resolved by a third reviewer. Data extraction was performed by 3 reviewers for the following variables:

(1) year of study (2) country of study (3) age range of the patients (4) the mean age in years (5) number of patients with male sex (6) number of patients with female sex (7) OAC size (8) OAC cause (e.g., extraction, trauma) (9) Intervention (PRF details: type, application method) (10) Comparator (e.g., buccal flap, collagen plug, no treatment) (11) Closure Rate (12) healing time (13) complications (14) primary outcome results (15) secondary outcome results; and (16) follow-up duration.

We included case reports and series to capture the full overview of initial clinical experience with PRF for OAC, as high-level comparative evidence is still emerging.

Any disagreements during the data extraction process were reviewed and resolved by two independent reviewers. To prevent data duplication, all extracted data were double checked for accuracy.

### Assessment of quality and bias risk

The methodological quality and risk of bias of the included studies were evaluated using tools appropriate to each study design. Randomized controlled trials were assessed using the **Cochrane Risk of Bias 2.0 (RoB 2)** tool (8). Non-randomized studies including retrospective, prospective, case-control, and cohort designs were evaluated using the **Methodological Index for Non-Randomized Studies (MINORS)** (9). Case reports and case series were assessed using the **Methodological Quality and Synthesis of Case Series and Case Reports tool** developed by Murad et al. (10).

### Meta-analysis of the included data

The meta-analysis was conducted using Review Manager (RevMan) version 5.4 (11). Dichotomous outcomes, including successful closure of oroantral communications or fistulas (OACs/OAFs), were analyzed by calculating risk ratios (RRs) with 95% confidence intervals (CIs). For studies reporting continuous variables, the mean difference was used as the summary measure. The analysis was performed using a random-effects model to account for variability across studies. Events and total sample sizes for each group were extracted from the included studies, and statistical heterogeneity was assessed using the Chi^2^ test and I^2^ statistic. Statistical significance was set at a *p*-value of < 0.05.

## Results

**Figure 1:**
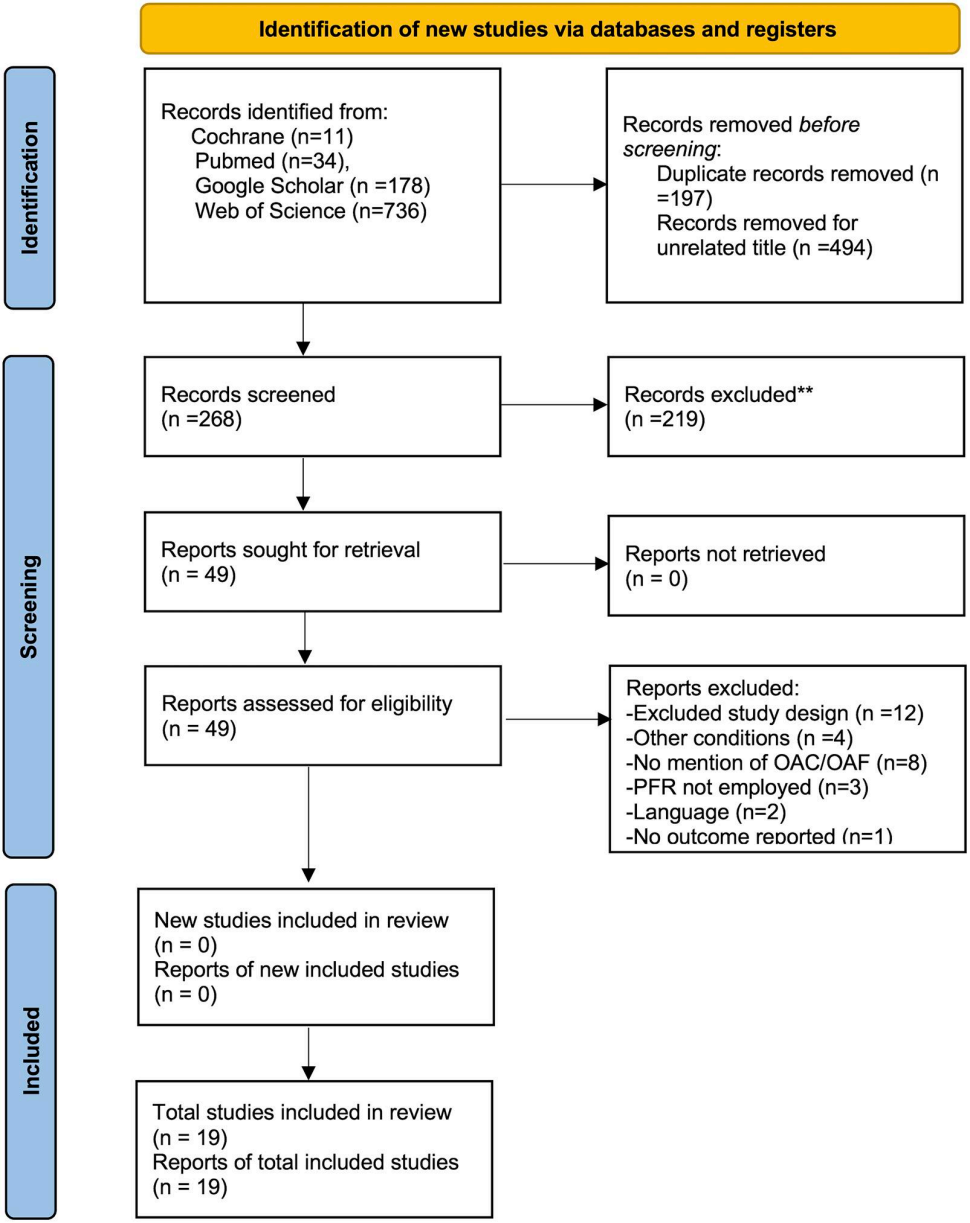
The preferred reporting items for systematic reviews and meta-analysis (PRISMA) flow chart.

Our systematic search yielded a total of 959 publications: 11 from Cochrane, 34 from PubMed, 178 from Google Scholar and 736 from Web of Science. After removing duplicates and studies with unrelated titles, 268 unique articles remained for abstract screening. Of these, 49 full-text publications were retrieved for detailed evaluation. Ultimately, 19 studies published between 2015 and 2025 met the inclusion criteria and were incorporated into the final analysis (Figure 1).

Among the included studies, six were case reports Mounzer, Al- Juboori, George, Pal, Hotta, Yang (12–17), and one case series Rebic (18). Seven studies were prospective cohort Elshourbagy, Elgabrty, Kapustecki, Bilginayar, Demetoglu, Bilginaylar, Nama (19–25), while two were retrospective cohort Esen, Orabona (26, 27). Three of the included studies were randomized controlled trial (RCT) Kaba, Hunger, Shukla (28–30). A summary of study characteristics is provided in the Supplementary Table S2.

In terms of country of origin, four studies were conducted in Turkey (21, 23, 26, 28), followed by two in Egypt (19, 25), two in India (15, 30), and two in the USA (14, 17). The remaining studies were conducted in Cyprus (22), Italy (27), Japan (16), Poland (20), Austria/Germany (29), Syria/ Yemen (12), Serbia (18), Iraq (24), and one was a multinational study involving Iraq, Saudi Arabia, and Brazil (13).

A total of 19 studies were included, with sample sizes ranging from 1 to 102 patients, and a combined total of 442 patients. The age of participants varied across studies, with reported ranges from 7 to 77 years. Mean ages were inconsistently reported; where available, they ranged between 34.6 ± 11.3 and 49 years. Gender distribution showed that male patients were slightly more represented overall, with several studies reporting higher male-to-female ratios. The most common cause of oroantral communication (OAC) was maxillary tooth extraction, specifically of molars or posterior teeth. Other less frequent causes included trauma, sinus lift complications, and odontogenic cysts. The size of the OAC varied considerably, with many studies describing defects greater than 3 mm in diameter, and some reporting sizes as large as 16 mm. Interventions primarily focused on the use of autologous platelet-rich fibrin (PRF), which was generally prepared by centrifuging the patient's own blood and applied as clots or membranes to the OAC site. Techniques varied, but most studies included suturing the PRF to surrounding tissues to prevent dislodgement. In some cases, PRF was combined with other materials such as bone grafts or used in conjunction with surgical procedures like buccal advancement flaps or sinus lifts. Comparators varied across studies. Seven studies included no formal comparator (i.e., PRF was used alone), while others used traditional methods such as buccal advancement flaps (22, 27, 29, 30), buccal fat pads, or alternative dressings such as oral wound dressings or sterile gauze (28). One study compared PRF to a 3D printed mesh (24), and another used a corticocancellous bone allograft as the comparator (17).

The closure rate reported in the included studies was consistently high across both treatment groups. In the majority of studies, complete closure was achieved in all patients, typically within 3 weeks postprocedure. Several studies reported full closure at 3-week follow-up and was also confirmed at 6 months in long-term follow-up for some studies. One study reported a closure rate of 90% in both treatment groups. In one study, 37.5% of patients in the PRF group achieved successful healing; however, the overall closure rate across all included studies approached nearly 100%.

No studies reported failure to achieve closure, except for minor variations in healing rates in specific subgroups. These results suggest that both interventions consistently achieved excellent closure rates across the included patient populations. Healing time varied across the included studies but demonstrated favorable and timely tissue and bone healing following the intervention. Epithelialized mucosa was consistently observed, in all patients,by the third week post-procedure. Granulation tissue formation was noted as early as day 7 or within the first week, followed by full mucosal epithelialization by week 3. Mucosa normalization in both color and texture was observed by 1 month in some studies, while radiographic bone healing was evident at approximately 3 months. Complete soft tissue healing was achieved within 2–4 weeks, with long-term follow-up confirming healing at 6 months through CBCT.

Healing durations ranged from 9 days to as long as 16 months in some complex cases. Bone density improvements were observed by 3 months in multiple studies. In summary, most patients achieved complete soft tissue healing within the first month, while bone healing continued progressively over several months. Overall, complications were rare and generally minor across all studies. Most studies (16 of 21) reported no complications.

Buccal sulcus obliteration (3 patients in two studies), persisting up to 12 weeks in one case. One study reported graft-stabilizing screw exposure in two patients and mobility of stabilizing elements in three patients, with one case involving localized inflammation. A few isolated cases of flap dehiscence and mesh exposure were also reported. No serious or life-threatening adverse events were observed. These findings demonstrate that the procedures were generally safe and well-tolerated, with minimal postoperative complications. The primary outcomes consistently demonstrated successful closure of oroantral communications with minimal complications. Complete closure of the defect was achieved in nearly all studies without recurrence. Several studies documented full closure of chronic OAFs without dehiscence, infection, allergic reactions, ulcerations, or graft exposure.

**Figure 2:**
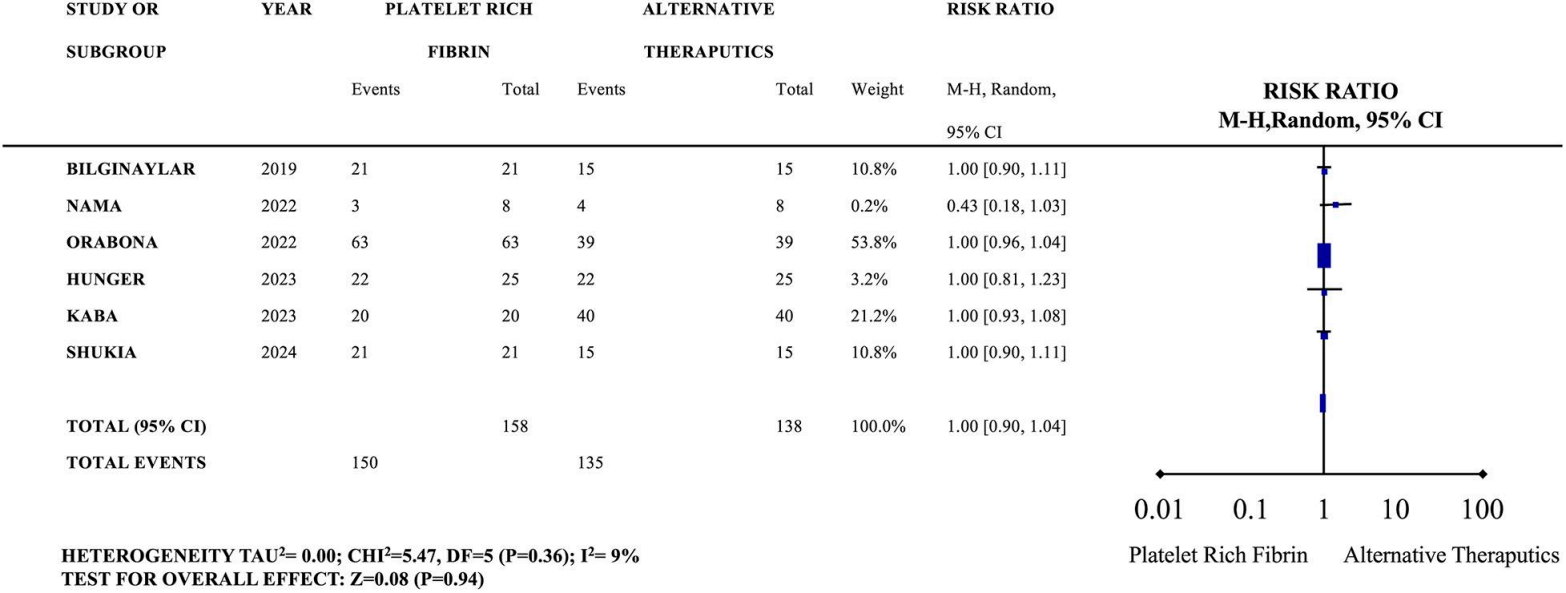
The forest plot chart comparing the closure success rate for the platlet rich fibrin therapy in compared to alternatice theraputics.

Patients treated with PRF experienced significantly better pain control (lower VAS scores), less analgesic use, and minimal swelling compared to buccal flap groups. Immediate and secure closure of the defect was typically achieved, with PRF effectively promoting rapid healing. Several studies compared surgical techniques, confirming the superiority of PRF and autogenous grafts in closure success. Smooth soft tissue recovery and full epithelialization were consistently reported. Overall, the included studies demonstrated high closure success rates, favorable healing outcomes, and minimal complications. The forest plot chart comparing the closure rate for the PRF therapy vs. alternatice theraputics showed acceptable heterogeneity (*I*^2^ = 9%). A very slight observed effect favored the alternatice z = 0.08, but this difference was not statistically significant (*p* = 0.94%) (Figure 2).

The secondary outcomes indicated additional clinical benefits particularly with the use of PRF. PRF offered similar surgical times compared to conventional methods but provided advantages such as preservation of sulcus depth, less invasiveness, greater comfort, and patient acceptability.

Bone density significantly improved by 3 months postoperatively, allowing for future implant placement. Histological findings showed new bone formation without inflammation or necrosis. PRF was associated with reduced morbidity and simplified surgical complexity. Multiple studies reported pain resolution, radiographic healing, improved mucogingival outcomes, and smooth soft tissue recovery. Temporary sensory disturbances and minor complications occurred infrequently. Fistula size, patient demographics, and minor complications were identified as factors influencing healing variability.

PRF consistently provided a safer, more predictable, and less invasive treatment option for OAC closure.

We assessed the methodological quality and risk of bias for all included studies. Case reports and case series (*n* = 8) were evaluated using the Murad et al. tool, which examines four domains: selection, ascertainment, causality, and reporting. Most studies fulfilled the criteria related to selection and ascertainment (10). However, none adequately addressed causality, and follow-up reporting was generally insufficient. Only three studies (Al-Juboori, Hotta, and Assad) met six or more of the eight quality items. Common methodological weaknesses included failure to rule out alternative causes and poor reporting of follow-up (Table 1).

**Table 1:** Bias assessment for the included studies according to the domains for evaluating the methodological quality of case reports and case series.

Domains for evaluating the methodological quality of case reports and case series								
	Selection	Ascertainment		Causality		Reporting		
Reference	Leading explanatory questions							
Al-Juboori et al. (13)	Q.1	Q.2	Q.3	Q.4	Q.5	Q.6	Q.7	Q.8
Hotta et al. (16)	Yes	Yes	Yes	No	No	Yes	Yes	Yes
Assad et al. (12)	Yes	Yes	Yes	No	No	Yes	Yes	Yes
Yang et al. (17)	Yes	Yes	Yes	No	No	Yes	Yes	Yes
Rebic et al. (18)	Yes	Yes	Yes	No	No	Yes	Yes	Yes
Bilginaylar (22)	Yes	Yes	Yes	No	No	Yes	Yes	Yes
George (14)	Yes	Yes	Yes	No	No	Yes	Yes	Yes
Pal et al. (15)	Yes	Yes	Yes	No	No	Yes	Yes	Yes

**Figure 3:**
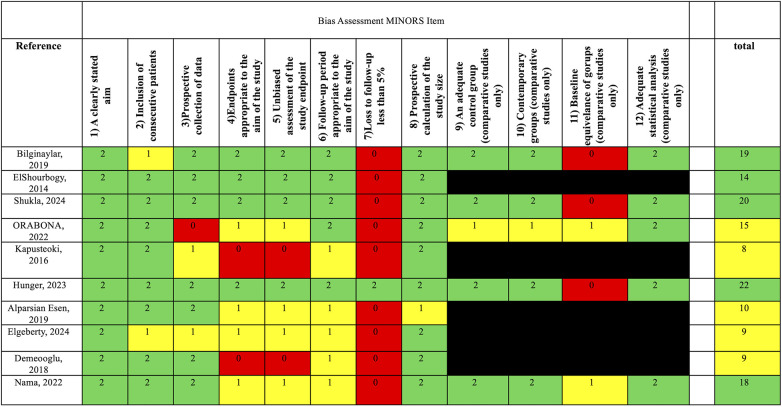
Methodological items for non-randomized studies (MINORS) assessment chart.

Non-randomized comparative studies (*n* = 10) were evaluated using the MINORS tool (9). Scores ranged from 8 to 22 out of 24. The highest scoring studies were Hunger et al. (29), Shukla et al. (30), and Bilginaylar (22), all scoring ≥19. Most studies clearly stated their aim and included consecutive patients, but frequent limitations included lack of prospective sample size calculation, incomplete follow-up, and inconsistent use of adequate control groups (Figure 3).

Three randomized controlled trial were included and assessed within the Cochrane RoB 2 tool framework as well (8) (Supplementary Table S3). Although it scored relatively high, limitations in bias control and statistical analysis were noted. Overall, only three studies were judged to have a low risk of bias, while the majority showed moderate to high risk, thereby limiting the overall strength and reliability of the synthesized evidence.

## Discussion

The utilization of platelet-rich fibrin (PRF) consistently demonstrates a safer, more predictable, and less invasive treatment option for closing oroantral communications (OAC) compared to alternative methods. Our systematic review aimed to assess the effects of platelet-rich fibrin therapy compared to alternative therapeutic modalities in individuals diagnosed with OACs or OAFS, regardless of age, gender, or etiology, in achieving successful closure of OACs/OAFS. Our review showed that PRF offered similar operative times to conventional methods but possessed several advantages, including sulcus depth preservation, reduced trauma (i.e., fewer teeth requiring extraction), greater comfort, and higher patient acceptance. In addition, many reports showed that pain resolved successfully, patients healed without complaint or significant delay, radiologically confirmed healing was achieved, and the soft tissue returned to its original condition.

The results of our systematic review align with previous studies on the safety and effectiveness of autologous platelet-rich fibrin (PRF) for oroantral communication (OAC) therapy. These observations were supported by Del Corso et al. (31, 32), where PRF demonestrated a successful closure rate higher than 95%.

In the majority of included studies, complete closure of mucosal ulcers occurred by 3 weeks, with radiographic bone healing evident by 3 months. Complications were rare and occasional minor ones, such as dehiscence of flap or transient obliteration of buccal sulcus. PRF was found to be more effective in our comparative review studies compared to more traditional treatments, such as gauze dressings or buccal advancement flaps. The benefits were better pain relief, faster wound healing, and improved patient comfort.

The findings of Castro et al. (32) were supported by the secondary outcomes of the studies analyzed, meaning that the PRF had the enhanced ability to accelerate mucogingival healing, decrease analgesic consumption, and reduce inflammation sequelae.

Histologic analysis showed the presence of new bone, which was not inflammatory or necrotic, consistent with the regenerative nature previously observed by Dohan Ehrenfest et al. (33). Our results contribute to growing evidence supporting PRF's safety and efficacy as a technique of closure, with advantages conventional techniques in terms of closure time, morbidity and patients satisfaction, making it an attractive alternative for both simple and complicated cases. This finding is supported by the systemic review of Dipalma et al. (34), who described PRF as an effective adjunct in the treatment of OAC/OAF. It failed to show any survival advantage for surgery. However, in their review, there were a few surgical techniques introduced, and PRF reportedly had regenerative capability with a low complication rate as well. Our study provides an advance on this work by providing the possibility to take a closer look at the clinical effectiveness of PRF, especially in minor and moderate-size defects.

Many previous surgical techniques focused solely on soft tissue closure without reconstructing the bony tissue, potentially affecting long-term stability of outcomes. Autologous bone transplantation is associated with longer operative times, increased bleeding, higher risk of infection, postoperative pain, and possible bone resorption over time. The follow-up period to assess bone regeneration remains limited, making it difficult to draw definitive conclusions about long-term results. Clinical experience with cryoplatelet gel remains preliminary and is based on a relatively small number of cases.

The biological process of bone reconstruction and stabilization can take a prolonged period (12–18 months). There is a lack of randomized controlled trials directly comparing bioengineered materials to traditional methods, limiting the strength of evidence. Current results are preliminary, and more rigorous, long-term studies are needed. Methodological limitations exist among included studies, many of which are case reports, case series, or observational with moderate to high risk of bias. There is significant heterogeneity in PRF preparation and application protocols, OAC defect sizes, and outcome assessment methods, complicating generalizability. Language barriers and access limitations may have led to exclusion of relevant studies. Potential publication bias is possible, as studies with positive results are more likely to be published. Most studies lacked control or comparison groups, which limits the ability to assess the relative efficacy of PRF in OAC closure.

It is important to mention that data from case reports/series indicate feasibility and preliminary safety but cannot support comparative efficacy claims and precludes strong causal inferences.

Future research should focus on conducting rigorously designed randomized controlled trials with sufficient sample sizes and standardized protocols for the preparation and application of platelet-rich fibrin. Extended follow-up periods are necessary to adequately assess the longterm durability and quality of bone regeneration and clinical outcomes. The inclusion of well-defined control or comparison groups is essential to robustly evaluate the relative efficacy of platelet-rich fibrin in oroantral communication closure. Researchers should adhere to strict methodological and reporting standards to minimize bias and improve reproducibility. Comprehensive analyses, including subgroup and sensitivity analyses, should be conducted to investigate sources of heterogeneity and factors affecting treatment success. International collaboration is encouraged to overcome language barriers and publication bias, ensuring a more inclusive evidence base. Finally, emphasis on patient-centered outcomes such as postoperative pain, functional recovery, and cost-effectiveness will better guide clinical decision-making and policy development.

## Conclusion

This systematic review synthesizes the avilable evidence the on autologous platelet-rich fibrin (PRF). The included studies showed high effectiveness of PRF in achieving closure for oroantral communications (OAC), with most studies reporting complete healing within 3–4 weeks. Based on this body of evidence, PRF appears to be a promising an potentially advantagous theraputic option. The included studies demonstrated numerous clinical benefits for PRF over traditional methods, including reduced postoperative pain, minimal complications, and improved mucosal and bone healing. Importantly, the addition of PRF also contributed to faster recovery, preservation of anatomical structures, and enhanced patient comfort making it a promising, minimally invasive alternative in OAC management.

The findings suggest that PRF not only supports soft tissue closure but also promotes bone regeneration, creating favorable conditions for future dental procedures such as implant placement. Due to the methodological heterogenity and overal low to moderate level of the current evidence, definitve conclusions need further investigation.

## Data Availability

The original contributions presented in the study are included in the article/Supplementary Material, further inquiries can be directed to the corresponding author.
