# Supplementary material for: Platelet-rich fibrin as a therapeutic modality for oroantral communication closure: a systematic review and meta-analysis
Source: Front Oral Health. 2026 Mar 11;7:1759248. doi: 10.3389/froh.2026.1759248 (PMC13013508; doi:10.3389/froh.2026.1759248)
Supplement: Supplementary file 1 [file Datasheet1.pdf]

Pubmed	("Oroantral communication" OR "Oroantral fistula" OR "Buccosinusal communication " OR "oroantral communications" OR "oroantral fistulas" OR "oroantral defect*" OR " oral antral communication*") AND ("Platelet-rich fibrin" OR "Platelet rich fibrin" OR PRF OR "Fibrin mesh" OR "fibrin s ealant" OR "autologous fibrin" OR "platelet concentrate")
Cochrane	#1 (Oroantral communication OR Oroantral fistula OR Buccosinusal communication O R "oroantral communications" OR "oroantral fistulas" OR "oroantral defect*" OR "oral antral communication*")<br>#2 ("Platelet-rich fibrin" OR "Platelet rich fibrin" OR PRF OR "Fibrin mesh" OR "fibri n sealant" OR "autologous fibrin" OR "platelet concentrate")<br>#3 (#1 AND #2)
Web of Science	=((("Oroantral communication*" OR "Oroantral fistula*" OR "Buccosinusal communication*" OR "oroantral defect*" OR "oral antral communication*")) AND (("Platelet-rich fibrin" OR "Platelet rich fibrin" OR PRF OR "Fibrin mesh" OR "fibrin sealant" OR "autologous fibrin" OR "platelet concentrate*")))
Google Scholar	("Oroantral communication" OR "Oroantral fistula" OR "Buccosinusal communication") AND ("Platelet-rich fibrin" OR PRF OR "Fibrin mesh")
