# Supplementary material for: Platelet-rich fibrin as a therapeutic modality for oroantral communication closure: a systematic review and meta-analysis
Source: Front Oral Health. 2026 Mar 11;7:1759248. doi: 10.3389/froh.2026.1759248 (PMC13013508; doi:10.3389/froh.2026.1759248)
Supplement: Supplementary file 2 [file Datasheet2.pdf]

Signalling questions	KABA 2023	HUNGER 2023	SHUKLA 2024
1.1 Was the allocation sequence random?	Y	Y	Y
1.2 Was the allocation sequence concealed until participants were enrolled and assigned to interventions?	Y	Y	Y
1.3 Did baseline differences between intervention groups suggest a problem with the randomization process?	N	N	N
Risk-of-bias judgement	Low	Low	Low
Optional: What is the predicted direction of bias arising from the randomization process?	Unpredictable	Unpredictable	Unpredictable

Signalling questions			Comments
2.1. Were participants aware of their assigned intervention during the trial?	PN	N	N
2.2. Were carers and people delivering the interventions aware of participants' assigned intervention during the trial?	Y	Y	Y
2.3. If Y/PY/NI to 2.1 or 2.2: Were there deviations from the intended intervention that arose because of the trial context?	Y	Y	Y
2.4 If Y/PY to 2.3: Were these deviations likely to have affected the outcome?	PN	PN	PN
2.5. If Y/PY/NI to 2.4: Were these deviations from intended intervention balanced between groups?	Y	Y	Y
2.6 Was an appropriate analysis used to estimate the effect of assignment to intervention?	PY	PY	PY
2.7 If N/PN/NI to 2.6: Was there potential for a substantial impact (on the result) of the failure to analyse participants in the group to which they were randomized?	-	-	-
Risk-of-bias judgement	Low	Low	Low
Optional: What is the predicted direction of bias due to deviations from intended interventions?	Unpredictable	Unpredictable	Unpredictable

Signalling questions	Comments		
2.1. Were participants aware of their assigned intervention during the trial?	N	N	PN
2.2. Were carers and people delivering the interventions aware of participants' assigned intervention during the trial?	Y	Y	Y
2.3. [If applicable:] If Y/PY/NI to 2.1 or 2.2: Were important non-protocol interventions balanced across intervention groups?	Y	Y	Y
2.4. [If applicable:] Were there failures in implementing the intervention that could have affected the outcome?	N	PN	N
2.5. [If applicable:] Was there non-adherence to the assigned intervention regimen that could have affected participants' outcomes?	N	N	N
2.6. If N/PN/NI to 2.3, or Y/PY/NI to 2.4 or 2.5: Was an appropriate analysis used to estimate the effect of adhering to the intervention?	-	-	-
Risk-of-bias judgement	Low	Low	Low
Optional: What is the predicted direction of bias due to deviations from intended interventions?	Unpredictable	Unpredictable	Unpredictable

Signalling questions	Comments		
3.1 Were data for this outcome available for all, or nearly all, participants randomized?	PY	Y	PY
3.2 If N/PN/NI to 3.1: Is there evidence that the result was not biased by missing outcome data?	-	-	-
3.3 If N/PN to 3.2: Could missingness in the outcome depend on its true value?	-	-	-
3.4 If Y/PY/NI to 3.3: Is it likely that missingness in the outcome depended on its true value?	-	-	-
Risk-of-bias judgement	Low	Low	Low
Optional: What is the predicted direction of bias due to missing outcome data?	Unpredictable	Unpredictable	Unpredictable

Signalling questions	Comments		
4.1 Was the method of measuring the outcome inappropriate?	N	N	N
4.2 Could measurement or ascertainment of the outcome have differed between intervention groups?	N	PN	N
4.3 If N/PN/NI to 4.1 and 4.2: Were outcome assessors aware of the intervention received by study participants?	PN	N	N
4.4 If Y/PY/NI to 4.3: Could assessment of the outcome have been influenced by knowledge of intervention received?	-	-	-
4.5 If Y/PY/NI to 4.4: Is it likely that assessment of the outcome was influenced by knowledge of intervention received?	-	-	-
Risk-of-bias judgement	Low	Low	Low
Optional: What is the predicted direction of bias in measurement of the outcome?	Unpredictable	Unpredictable	Unpredictable

Signalling questions			Comments
5.1 Were the data that produced this result analysed in accordance with a pre-specified analysis plan that was finalized before unblinded outcome data were available for analysis?	PY	Y	Y
Is the numerical result being assessed likely to have been selected, on the basis of the results, from...			
5.2. ... multiple eligible outcome measurements (e.g. scales, definitions, time points) within the outcome domain?	N	N	N
5.3 ... multiple eligible analyses of the data?	N	N	N
Risk-of-bias judgement	Low	Low	Low
Optional: What is the predicted direction of bias due to selection of the reported result?	Unpredictable	Unpredictable	Unpredictable
